# Preserving the Cellular Tissue Structure of Maize Pith Though Dry Fractionation Processes: A Key Point to Use as Insulating Agro-Materials

**DOI:** 10.3390/ma14185350

**Published:** 2021-09-16

**Authors:** Claire Mayer-Laigle, Laia Haurie Ibarra, Amélie Breysse, Marina Palumbo, Frédéric Mabille, Ana Maria Lacasta Palacio, Cécile Barron

**Affiliations:** 1 IATE, University of Montpellier, INRAE, Institut Agro, 34060 Montpellier, France; amelie.breysse@hotmail.fr (A.B.); frederic.mabille@inrae.fr (F.M.); cecile.barron@inrae.fr (C.B.); 2 GICITED Research Group, Departament de Tecnologia de l’Arquitectura, Universitat Politècnica de Catalunya, 08034 Barcelona, Spain; laia.haurie@upc.edu (L.H.I.); mariana.palumbo@upc.edu (M.P.); ana.maria.lacasta@upc.edu (A.M.L.P.)

**Keywords:** plant tissues, milling, electrostatic separation, thermal properties, gravity sorting

## Abstract

Plant biomass has various compositions and structures at different scales (from the component organs to their constitutive tissues) to support its functional properties. Recovering each part of the plant without damaging its structure poses a challenge to preserving its original properties for differential dedicated end uses, and considerably increases its added value. In this work, an original combination of grinding based on shearing stress and separation based on particle size and density was successfully used to sort rind (65% *w*/*w*) and pith (35% *w*/*w*) from maize stem internodes. More than 97% of the rind was isolated. The pith alveolar structure was well preserved in coarse particles, making them suitable for insulation bio-based composite materials, a promising alternative to conventional nonbiodegradable insulation panels. Boards produced from the dry fractionated pith exhibited thermal conductivities like those produced from hand dissected pith, with values equal to 0.037 W·mK^−1^ and 0.039 W·mK^−1^, respectively. In the finest fraction (particle size <1 mm), the pith vascular bundles (around 300–400 µm in diameter) were dissociated from parenchyma cells and successfully isolated using a cutting-edge electrostatic separator. Their structures, which provide the plant structural support, make them potentially valuable for reinforcement in composite materials.

## Highlights

- Pith and rind were isolated from maize stem after knife milling and density sorting
- Vascular bundles were sorted using electrostatic separation
- Shear stress during grinding preserves the cellular structure of maize pith in the particles
- Insulating boards made with maize pith exhibit thermal conductivities similar to conventional insulating materials
- Pith particles isolated by dry fractionation processes keep their thermal insulative properties

## 1. Introduction

Fossil fuels have an unsustainably huge greenhouse gas footprint, and it is essential to find alternative solutions. The efficient use of natural resources, including reducing postharvest losses as petrol replacer, are among the United Nations sustainable development goals (SDGs) [1]. In particular, the use of lignocellulosic materials as an alternative to fossil feedstock for chemistry, energy, and materials is a target for considerable research efforts [2,3,4]. Part of the way forward revolves around the development of biorefining to produce biomolecules, bioenergy, and agromaterials [5]. One example is the increasing demand on thermal insulation materials in building applications. In Europe, the global demand is projected to increase at a compound annual growth rate of 4.5% between 2016 and 2027 [6]. However, the use of biobased materials for such purposes is not yet widespread, and many research efforts are still needed to propose alternative insulating materials [7].

The conversion of lignocellulosic biomass from forest residuals, crop residues, and energy crops is of special interest as it offers an unlimited reservoir of functional components that do not add to competition for food [8,9]. Plant biomass has different compositions and structures at different scales (from the component organs to their constitutive tissues) to support different functional properties (e.g., mechanical support, protection from attacks or water, nutrient storage and transport, and so on [10]). A full biorefinery scheme designed to optimize the biomass material flows could exploit these intrinsic properties to differentiate end uses [11].

The maize stem is a part of maize stalk, which is an important crop residue left after maize grain harvest [12]. Its inner part (the pith) has an alveolar structure [13] much like expanded polystyrene that could be exploited in insulative composite materials as it has been proposed for sunflower stalks [14], maize cob [15,16,17], and maize stalk [18,19,20,21]. Boards produced from crop pith are a promising alternative to conventional nonbiodegradable insulation panels from fossil origin, but its extraction processes must keep its native properties and should be easily industrialized. The vascular bundles of the pith and its surrounding anatomical component called the rind provide to the plant structural mechanical support, which makes them potentially valuable for reinforcement in composite materials, but not for bioenergy applications, as they are not readily hydrolysable [22]. Mild deconstruction of the plant material at the relevant tissue scale (100 to 500 µm) followed by sorting steps could be a potential route to separate each part of the maize stem into dedicated end use streams, provided that the process used preserves the structure of each tissue on which their properties are based.

In this work, the potential of dry fractionation processes combining milling and physical separation was explored to separate the different parts of maize stem without damaging their cellular structures. The aim was to conserve the specific properties of each part to dedicate them to a specific end use: insulating materials for the pith, and reinforcement in agrocomposite materials for the rind and vascular bundles. In this original approach, based on reverse engineering, the structure of the plant materials and the properties targeted in the end product drive the choice of the processes used. The efficiency of dry fractionation processes has already been demonstrated on various other lignocellulosic biomasses [23,24,25], but not yet on maize where the foam structure of the pith makes it particularly delicate to fractionate in a mill without crushing it. We first focused on sorting the pith and the rind from the maize internode; the main challenge being to find a suitable milling technology to dissociate the two tissues without modifying their structures. In practice, the grinder transmits the mechanical load to the raw materials through a combination of different loading modes that can be compression, impact, shearing, or abrasion/attrition [10]. The proportion of each mode depends on the technologies employed and the process parameters. The way the forces are applied and transmitted to the matter in the milling chamber dictates the propagation of the fracture path, which in turn affects the properties of the resulting particles. For instance, size-reduction technologies (i.e., milling) that enhance impact rather than shear can preserve the alveolar structure of cork and the resulting mechanical properties of the resulting particles [26]. On the contrary, for flax fibre, the compression and impact generated by severe ball milling leads to the destruction of the fibre and a drastic comminution, whereas shear allows a defibrillation of the fibre with a weak particle size reduction [27]. Thus, the relation between the loading mode employed and the structure of the plant materials is paramount, and the choice of the reliable technology must be based on a deep knowledge of the plant materials’ properties.

The structure of maize was first studied though dissection and SEM observation, and adapted milling technologies were proposed. The influence of the milling process on the structure of the plant materials and the physical properties of the ground powder was studied. An appropriate sorting step based on the difference in size and density observed in the ground particles was used to separate and recover each of the plant material tissues. To go further in the separation, we also demonstrated that an innovative technique based on electrostatic phenomena, exploiting the difference in density and shape of the particles, enables an efficient separation for isolating vascular bundles from the pith, opening new valuable perspectives for this part of the pith which had previously brought no added value for insulating board. Finally, to confirm the potential of the processes developed in the industrialization of insulating biobased materials, insulating boards prepared with the pith fractions obtained through dry fractionation processes have been produced. Their thermal properties have been studied and compared to those obtained on boards made of pure pith extracted by hand dissection.

## 2. Material and Methods

### 2.1. Materials

Maize stems were obtained as crop residue left after maize grain harvest (Maugio, France, UMR Diascope). The maize stem internodes were manually isolated and cut into 2-cm-long rods ready for fractionation. Proportions by weight of pith and rind were obtained after hand dissection.

### 2.2. Dry Fractionation Processes

**Figure 1:**
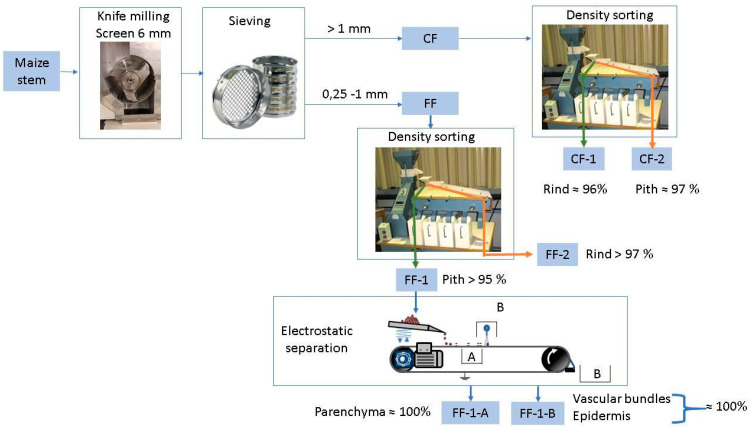
Process flow diagram for sorting the pith-dense fraction (FF-1-A and CF-1), rind-dense fraction (FF-2 and CF-2), and vascular bundles (FF-1-B).

Several devices were employed for the milling and sorting of the different maize fractions (Figure 1).

*Milling:* Milling was performed using a SM100 model knife mill (Retsch, Eragny, France) equipped with a 6 mm sieving mesh operated at a rotor speed of 1500 rpm.

*Sieving:* Sieving was performed in a sieving column (ROTEX, Villeneuve la Garenne, France) equipped with two sieves (1 mm and 0.25 mm mesh size screens). A 50 g amount of sample was sieved for 10 min by rotary motion. The coarse fraction (CF) was recovered on the 1-mm sieve; and the fine fraction (FF) was recovered between the 0.25-mm and 1-mm sieves.

*Density sorting:* A small-scale seeds LA-K model gravity separator (Westrup, Slagelse Denmark) was used to separate the powder on the basis of particle density. This device combines an air bed technology and an oscillating triangular grading deck that separates and moves the particles to one side or the other according to their size, weight, and density. Airflow was set to the minimal value, and the table was set to a gentle incline. Two opposite fractions were recovered on the left (FF-1 and CF-1) and right (FF-2 and CF-2) of the table.

*Electrostatic separation:* Electrostatic separation was performed using a lab-prototype corona belt separator (see [28] for details) that can be seen as a smaller-scale version of the electrostatic drum separator widely used in plastics and minerals processing industries. The voltage of the corona discharge was set to 25 kV, and the sorted particles were collected in three places: (i) on the side of the conveyor near the electrode for the particles blown by the ionic wind generated during the discharge (fraction FF-1-A), (ii) on the support housing the electrode generating the corona discharge, and (iii) at the end of the conveyor. The fractions collected at ‘(ii)’ and ‘(iii)’ were pooled as the fraction FF-1-B.

### 2.3. Particle Characterization

*Optical microscopy*. Cross-sections of prehydrated maize internodes (thickness 200 µm) were cut using a vibratome (HM650V Microm, Walldorf, Germany) then observed as particles using an AZ100M multizoom microscope (Nikon, Tokyo, Japan) with white LED epi-illumination at low magnification (x2).

*Scanning electron microscopy (SEM).* Samples were stuck onto carbon adhesive tape and observed directly, without any metal sputter coating, using a benchtop Phenom Pro X scanning electron microscope (Phenom World, Eindhoven, The Netherlands) with an acceleration voltage of 10 kV in image mode and a backscattered electron detector.

*Quantification of the purity of the fraction.* Proportions of pith, rind, and vascular bundles were determined by manual particle counting after visual identification of about 100 particles for each sample.

### 2.4. Preparation of Insulating Thermal Boards and Thermal Characterization

Insulating thermal boards were prepared by mixing the maize pith fractions with an alginate binder in a proportion of 5% wt. The boards were compacted during 10 min and afterwards dried at 40 °C. For this study, three maize pith fractions were specifically produced and tested. The first one, called “Manual_pith”, is considered the most native pith fraction as it was manually extracted by hand dissection of the stem. The pith fragments were subsequently chopped with a knife mill and gravity sorted by keeping the fraction retained between 1 and 4 mm. The two others fractions were obtained through the dry fractionation process previously described, but slightly adapted to the raw material. A dry fractionated medium pith fraction (DF_medium) was obtained after gravity sorting of the 1–2 mm sieved fraction from knife milling (using a 10 mm sieving mesh), and a dry fractionated coarse pith fraction (DF_coarse) was obtained after gravity sorting of the >2 mm sieved fraction from knife milling. Board thermal properties were measured with a Quickline-30 Thermal Properties Analyser using a surface probe with a disk sensor. Such equipment is based on the analysis of the transient temperature response of the material to heat flow induced by electrical heating. Prior to the test, the samples have been conditioned at 20 °C and 60% moisture content for one week.

## 3. Results and Discussion

### 3.1. Pith and Rind Isolation from Maize Stem

**Figure 2:**
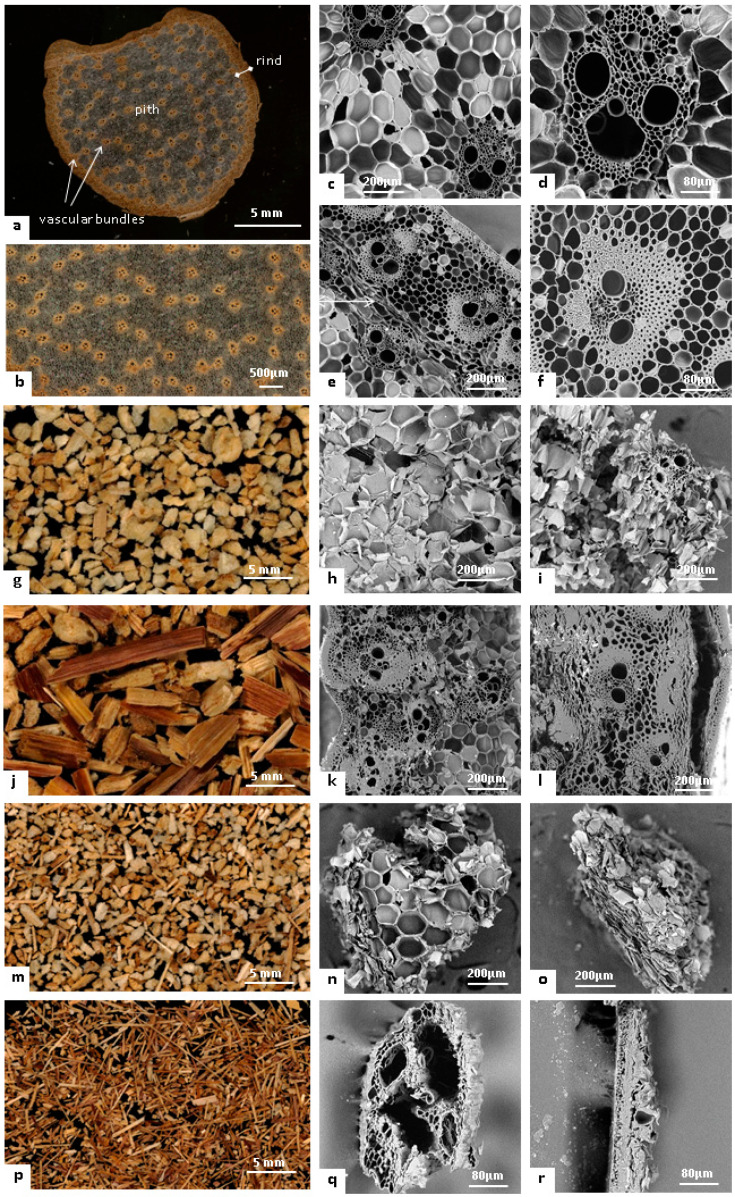
Visualization of maize stem internode structure (**a**–**f**) and fractions isolated: CF-2 (**g**–**i**), CF-1 (**j**–**l**), FF-1-A (**m**–**o**) and FF-1-B (**p**–**r**). **a**,**b**: Optical microscopy of maize internode cross-section; (**g**,**j**,**m**,**p**): optical microscopy of isolated fractions CF-2, CF-1, FF-1-A, and FF-1-B, respectively; c,d: SEM microscopy of pith structure in the maize internode; (**e**,**f**): SEM microscopy of rind structures in the internode pith, **h**,**i**: SEM microscopy of section CF-2 particles; (**k**,**l**): SEM microscopy of section CF-1 particles; (**n**,**o**): SEM microscopy of section FF-1-A; (**q**,**r**): SEM microscopy of section FF-1-B particles.

The maize stem internode is composed of two main regions, i.e., pith and rind (Figure 2a,b), that account for 35% and 65% by weight, respectively, in the internode as previously reported [29]. Visible throughout the pith under optical and SEM microscopy are vascular bundles (including both conducting tissues, xylem and phloem, surrounded by sclerenchyma cells, Figure 2d) spread into parenchyma cells (Figure 2b,c). This part of the stem forms rigid tubular ‘fibre-like’ structures that lie in the axis of the stem, embedded in an isotropic solid honeycomb foam structure. The apparent density of the pith is around 85 kg·m^−3^. The proportion by weight of this ‘fibrous’ structure is about 35% of the pith, i.e., 12% of the total maize internode, as also observed for sorghum stems [30]. The rind (Figure 2e,f) is packed with vascular bundles and sclerenchyma cells surrounded by an epidermis on the most external part that account for about 15% by weight of the rind. The rind, appearing as the brown part of the internode, is characterized by a denser (apparent density around 250 kg·m^−3^) anisotropic structure with smaller cortical parenchyma cells, vascular bundles including a bigger sclerenchyma sheath made of smaller cells, and generally more lignified cell walls [31].

The idea behind this work was to dissociate the different tissues while preserving their cellular structure, assuming that this is mandatory for its industrial use. We assumed that impact-mode mechanical loading would compact the pith, while shear-mode mechanical loading would propagate the fracture path between the different tissues due to their different mechanical properties [32]. Thus, we chose to use knife milling, which mainly works by shear-mode mechanical loading, as it has produced similar histological-scale dissociation patterns on other lignocellulosic biomasses, including Douglas fir bark [33], flax shives [34], and sunflower stalks [14]. We worked on the assumption that pith particles had to contain at least 10 parenchyma cells (around 150 µm in diameter/each as observed in Figure 2c) to maintain their honeycombed foam structure. We therefore performed a coarse milling by employing a 6 mm sieve in the continuous-drive milling device. The ground powder had a wide particle-size distribution, with particles ranging from 0.1 mm up to 20 mm with differing shapes and colours, and large numbers of elongated brownish-colour particles (from the rind) comingled with round whitish-colour particles (pith). Some of the particles exhibited both colours on different faces. Pure particles of pith and rind were therefore easily identifiable, validating the assumptions made for the choice of milling technology. Density sorting was investigated to separate pith particles from rind particles based on their density difference. As density sorting efficiently separates particles of same size range, we applied a preliminary sieving step to class particles into two fractions: a coarse fraction (CF; 65% by weight), and a fine fraction (FF; 30% by weight). Particles passing through the holes of the 0.25 mm sieve (5% by weight) were considered as dust and not sorted on the gravity separator. Fractions recovered on the extreme left of the table (FF-1 and CF-1) were composed of the lighter particles that had been slightly deviated by the vibrations, whereas fractions recovered on the extreme right of the table were composed of denser particles (FF-2 and CF-2). Based on the apparent density of maize tissues within the stem, rind particles were up to three times more dense than pith particles. CF-1 was mainly composed of whitish particles (Figure 2g) that were identified as pith particles presenting the typical honeycombed parenchyma structure (Figure 2h), including some vascular bundles (Figure 2i). Parenchyma cells were slightly compressed in some of the particles (Figure 2l), but on the whole, the initial honeycomb structure was well preserved. On the other side, the CF-2 fractions were mainly elongated brown-coloured particles (Figure 2j). Cross-sections of these particles observed by SEM (Figure 2k,l) exhibited the typical rind structure composed of dense vascular bundles spread into cortical parenchyma with a still relatively adherent epidermis. CF-1 and CF-2 had more than 95% purity in both pith and rind. The FF-1 and CF-1 fractions weighed substantially less than the FF-2 and CF-2 fractions due to the large density difference between pith and rind. The intermediate-purity fractions recovered in the middle of the deck (approximately 20% by weight) needed to be recirculated to increase their purity. Density sorting of CF and FF allowed recovery of more than 97% of the rind in the CF-2 and FF-2 fractions. CF-1 and FF-1 contained about a third of the total pith from the internode, which is a relatively low yield. This low yield is explained by the fact that a large proportion of pith particles was captured in the intermediate fractions from the gravity separator that needed to be recirculated. Some pith particles were also captured in the gravity separator airflow or recovered in the finest fraction (dust).

### 3.2. Isolation of Vascular Bundles

The FF-1 fraction mostly composed of lighter particles also contained elongated white or brown particles that were sorted with a corona electrostatic separator (Figure 1). Given the lower size of these particles, we assumed that the pith had split into parenchyma cells and vascular bundles. Some particles got blown in the ionic wind created by the corona discharge and were recovered on the side of the conveyor (FF-1-A, Figure 2m,n,o). These light-coloured particles exhibited near-cubic shapes (Figure 2m). Under SEM, these whitish particles exhibited the typical parenchyma cell structure (Figure 2n,o) without vascular bundles. However, they also appeared visibly more compressed than pith particles extracted in CF-1. These smaller-sized particles may have undergone more compaction during the milling step. FF-1-A showed very high purity, with rates approaching 99% in parenchyma. The most elongated particles from the FF-1 fraction got polarized and were either attracted by the corona discharge electrode support (in PVC) or else not deviated and recovered at the end of the conveyor (FF-1-B, Figure 2p). These particles were identified as vascular bundles (Figure 2q) or pieces of epidermis (Figure 2r), explaining, thus, the brownish colour of the FF-1-B fraction (Figure 2p). The vascular bundles obtained were around 300–400 µm in diameter and contained a thin sclerenchyma sheath, pointing to the pith as origin. As the chemical compositions of pith and rind are similar, in particular for the main cell wall constituents [35,36], the histological separation during the electrostatic sorting was mainly explained by the different morphological responses of particles to the corona discharge.

Vascular bundles from the rind were not isolated in this study. However, the strong adherence between cortical parenchyma cells and sclerenchyma sheath, as suggested by the higher lignification of these tissues [37], could explain why fracture depth was not located in this region. Further milling of the rind pieces could be helpful to isolate such structures if they have differences in physical properties within the rind.

### 3.3. Insulation Materials from Pith Tissue: Impact of Isolation Procedure

**Figure 3:**
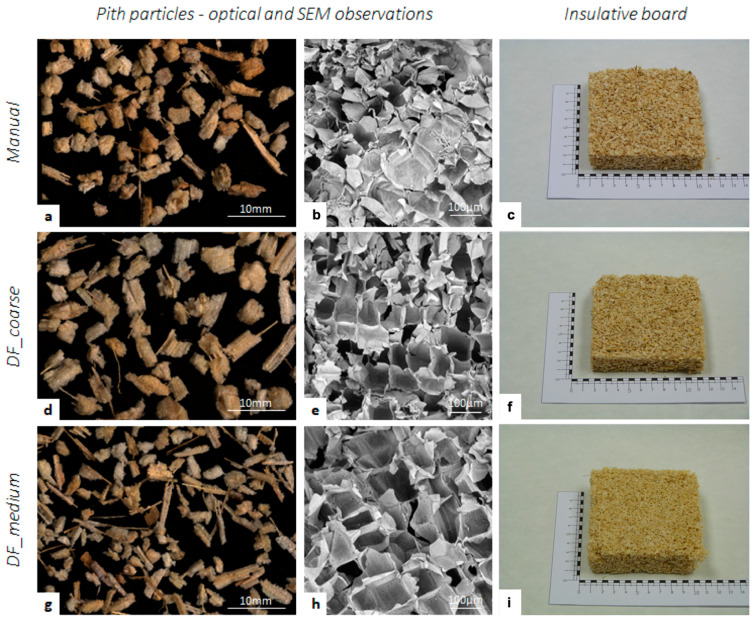
Visualization of pith particles isolated manually (**a**,**b**) or by dry fractionation process (‘DF_coarse’: **d**,**e**; ‘DF_medium’: **g**,**h**) and insulative board prepared with these pith fractions (**c**,**f**,**i**). Optical microscopy of isolated fractions (**a**,**d**,**e**), SEM microscopy o section of pith particle from each pith fraction (**b**,**e**,**h**); Insulative board prepared from each fraction (**c**: manual pith fraction, **f**: ‘DF-coarse’ pith fraction, **i**: ‘DF_medium’ pith fraction).

Alveolated structure of pith tissue could be of interest to produce thermal insulating materials, as already demonstrated in the sunflower stalk valorisation [14] or maize residues [16,18]. The potential interest of extracting pith from maize with an industrializable process was tested, as well as the eventual impact of the isolation process. Three pith fractions were produced to prepare insulation boards whose thermal properties were measured. One manually extracted pith tissue was used as the reference, and two fractions were isolated using a fractionation diagram as proposed previously. Two fractions differing in medium particle size (DF_coarse and DF_medium) were produced in order to check if the structure of the finest particles, that could be more compressed, was still appropriate (Figure 3). As already observed, particles originating from pith are round and whitish-coloured. Some linear structures can be observed on the edge and were assigned to the vascular bundles from the pith (Figure 3a,d,g). Some brown particles with flat shapes were observed in dry fractionated samples due to contamination by some particles originating from the rind. These two fractions were highly enriched in pith but were not, strictly speaking, pure pith. Manually extracted pith fraction had an intermediate particle size, in between the DF_coarse and DF_medium fractions. Open honeycomb alveolar structure was pointed out by SEM observation of sectioned pith particles (Figure 3b,e,h), regardless of the samples. Even for the smallest particles, no compaction of parenchyma cell was evidenced, and this could be related to the shear-mode mechanical loading in the knife mill.

Thermal insulating boards were then prepared from these three pith fractions, using the same preparation process and amount of binder. The pressure applied during the preparation resulted in less compression on the sample with finest particle size (DF_medium), which led to a lower density in this board (Table 1). We could also observe better cohesion in the Manual_pith and DF_coarse boards. However, the difference in density did not significantly affect the values of thermal conductivity (λ). The dry fractionation process did not damage the pith structure, at least in terms of thermal properties, which is reflected in the similar values obtained for Manual_pith and DF_coarse boards (Table 1). These thermal properties, together with the possibility to establish an industrialized production method, could allow the biobased insulation materials from crop by-product to be a sustainable alternative to nonrenewable insulation materials such as expanded polystyrene or foamed polyurethane.

## 4. Conclusions

The maize stem internode is essentially composed of two main components, the pith (35% *w*/*w*) and the rind (65% *w*/*w*), that exhibit different cellular structures. Pith particles form a foam-like structure, and vascular bundles from the pith can be considered as fibrous materials. Rind particles are also rigid and fibrous and have potential as a reinforcement in composite materials. Here, for the first time, a judicious combination of knife milling, density sorting, and electrostatic separation was used to successfully isolate each botanical part of the maize stem without altering the cellular structures. 97% of the rind fraction was thus isolated. This original mild deconstruction at the tissue scale (100 to 500 µm) preserved the histological structure of the plant, as the initial honeycomb pith structure was still observed after mechanical extraction in SEM images. Biobased insulation boards prepared with fractionated pith showed thermal properties (λ = 0.037 W mK^−1^) similar to the ones obtained with manual pith extraction (λ = 0.037 W mK^−1^), which indicates that pith structure was not affected by the mechanical fractionation process. However, the size of pith particles has an effect on the materials’ preparation and properties and needs to be further optimized. Mild deconstruction via a dry fractionation process thus opens up huge perspectives to fully exploit the native plant structures. Now, further investigations are needed to scale this isolation procedure up to a kilogram-scale process in order to yield fractions testable for functional properties and new dedicated end uses. Moreover, further developments will be also investigated to extract pure pieces of hydrophobic structures such as epidermis or isolate fibrous and highly lignified structures from the maize rind that can be potentially valuable for reinforcement in composite materials.

## Figures and Tables

**Table 1:** Density and thermal properties of insulating boards prepared with three maize pith fractions either manually extracted (“manual pith”) or obtained after dry fractionation process (‘DF_coarse’ and ‘DF_medium’).

	Manual_Pith	DF_Coarse	DF_Medium
Density (kg·m^−3^)	37	33	23
Thermal conductivity λ (Wm·K^−1^)	0.039	0.037	0.035
Volumetric heat capacity α (J m^−3^·K^−1^)	0.953 × 10^−6^	0.916 × 10^−6^	1.115 × 10^−6^
c·ρ (m^2^·s^−1^)	0.040 × 10^6^	0.040 × 10^6^	0.031 × 10^6^

## Data Availability

Data sharing is not applicable to this article.

## Nomenclature (See Figure 1)

CF	Coarse fraction obtained from knife milling (6 mm sieving mesh) and sieving (>1 mm)
CF_1	Dense fraction obtained from gravity sorting of the coarse fraction (CF)
CF_2	Light fraction obtained from the gravity sorting of the coarse fraction (CF)
DF_Coarse	Dry fractionated coarse pith fraction obtained after gravity sorting of the >2 mm sieved fraction from knife milling (10 mm sieving mesh).
DF_Medium	Dry fractionated medium pith fraction obtained after gravity sorting of the 1–2 mm sieved fraction from knife milling (10 mm sieving mesh)
FF	Fine fraction obtained from knife milling (6 mm sieving mesh) and sieving (0.25–1 mm)
FF-1	Dense fraction obtained from gravity sorting of the fine fraction (FF)
FF-2	Light fraction obtained from the gravity sorting of the fine fraction (FF)
FF-1-A	Fraction deviated by the ionic cloud during the electrostatic sorting of the fine fraction 1 (FF-1)
FF-1-B	Fraction not deviated by the ionic cloud during the electrostatic sorting of the fine fraction 1 (FF-1)
Manual_Pith	Manually extracted fraction, by hand dissection of the stem
SEM	Scanning Electron Microscopy
